# Total Usual Micronutrient Intakes Compared to the Dietary Reference Intakes among U.S. Adults by Food Security Status

**DOI:** 10.3390/nu12010038

**Published:** 2019-12-22

**Authors:** Alexandra E. Cowan, Shinyoung Jun, Janet A. Tooze, Heather A. Eicher-Miller, Kevin W. Dodd, Jaime J. Gahche, Patricia M. Guenther, Johanna T. Dwyer, Nancy Potischman, Anindya Bhadra, Regan L. Bailey

**Affiliations:** 1 Interdepartmental Nutrition Program, Purdue University, 700 W. State Street, West Lafayette, IN 47907, USA; cowan9@purdue.edu (A.E.C.); jun24@purdue.edu (S.J.); heicherm@purdue.edu (H.A.E.-M.); 2 School of Medicine, Wake Forest University, 475 Vine St, Winston-Salem, NC 27101, USA; jtooze@wakehealth.edu; 3 NIH National Cancer Institute, 9609 Medical Center Drive, Rockville, MD 20850, USA; doddk@mail.nih.gov; 4 NIH Office of Dietary Supplements, 6100 Executive Blvd., Bethesda, MD 20892, USA; jaime.gahche@nih.gov (J.J.G.); dwyerj1@od.nih.gov (J.T.D.); potischn@mail.nih.gov (N.P.); 5 Department of Nutrition and Integrative Physiology, University of Utah, 250 South 850 East, Salt Lake City, UT 84112, USA; PMGuenther@outlook.com; 6 Jean Mayer USDA Human Nutrition Research Center on Aging, Tufts University, 711 Washington Street, Boston, MA 02111, USA; 7 Department of Statistics, Purdue University, 250 N. University St, West Lafayette, IN 47907, USA; bhadra@purdue.edu

**Keywords:** NHANES, dietary supplement, micronutrients, DRI, food security

## Abstract

This study examined total usual micronutrient intakes from foods, beverages, and dietary supplements (DS) compared to the Dietary Reference Intakes among U.S. adults (≥19 years) by sex and food security status using NHANES 2011–2014 data (*n* = 9954). DS data were collected via an in-home interview; the NCI method was used to estimate distributions of total usual intakes from two 24 h recalls for food and beverages, after which DS were added. Food security status was categorized using the USDA Household Food Security Survey Module. Adults living in food insecure households had a higher prevalence of risk of inadequacy among both men and women for magnesium, potassium, vitamins A, B6, B12, C, D, E, and K; similar findings were apparent for phosphorous, selenium, and zinc in men alone. Meanwhile, no differences in the prevalence of risk for inadequacy were observed for calcium, iron (examined in men only), choline, or folate by food security status. Some DS users, especially food secure adults, had total usual intakes that exceeded the Tolerable Upper Intake Level (UL) for folic acid, vitamin D, calcium, and iron. In conclusion, while DS can be helpful in meeting nutrient requirements for adults for some micronutrients, potential excess may also be of concern for certain micronutrients among supplement users. In general, food insecure adults have higher risk for micronutrient inadequacy than food secure adults.

## 1. Introduction

The 2015–2020 Dietary Guidelines for Americans (DGA) reported a number shortfall nutrients among U.S. adults including calcium, iron, magnesium, potassium, choline, folate and vitamins A, C, D, and E [1,2]. The DGA also recognized food insecurity, defined as limited availability of foods and an individual’s inability to access food [3], as a potential barrier to a healthy diet that warrants further research [1]. Indeed, a systematic review concluded that food insecure adults have lower intakes of vitamin A, vitamin B6, calcium, magnesium, and zinc from diet alone when compared to those who were food secure [4]. Dietary supplement (DS) use is also lower in adults living in food insecure U.S. households than in those that are food secure [5], implying that differences in micronutrient intakes between the food secure and food insecure population subgroups might be amplified when total nutrient intakes, inclusive of DS, are considered; however, to our knowledge, no study has compared total usual nutrient intakes by household food security status. Therefore, the purpose of this analysis was to estimate the prevalence of risk of micronutrient inadequacy and excess by comparing total usual micronutrient intake distributions to the Dietary Reference Intakes (DRI); and to parse out the contributions of DS to the total intakes of U.S. adults (1) in the general population and (2) by household food security status, using data from the National Health and Nutrition Examination Survey (NHANES), 2011–2014.

## 2. Methods

The NHANES is a nationally representative, continuous cross-sectional survey of noninstitutionalized, civilian residents of the U.S. conducted by the National Center for Health Statistics. Complete details of the NHANES survey are publicly available [6]. Briefly, the NHANES protocol includes an in-person household interview that queries health information and demographics as well as a follow-up health examination in a Mobile Examination Center (MEC) for each participant. Written informed consent was obtained for all participants or their proxies, and the NHANES protocol (and publicly released de-identified data) was approved by the Research Ethics Review Board at the CDC/National Center for Health Statistics. For the purposes of this analysis, the most recent data on dietary and DS intakes available from the NHANES (2011–2012 and 2013–2014 cycles) were combined to form an analytic sample of 19,151 participants. These survey years were combined in order to increase the statistical reliability of estimates across population subgroups [6]. Participants who were <19 years of age (*n* = 7939), did not complete or had incomplete 24 h dietary recall or dietary supplement questionnaire data (*n* = 1088), or who were pregnant and/or lactating (*n* = 170) were excluded, yielding a final analytic sample size of 9954 adults.

All demographic data used for this analysis were collected from participants in NHANES using the Computer-Assisted Personal Interview system during the household interview. Household food security status was measured using the USDA’s Household Food Security Survey Module; one household reference person responded to 18 items for households with children, or 10 items for households without children. The USDA’s Household Food Security Survey Module is on a continuum comprised of four different food security classifications, ranging from full, marginal, low, to very low household food security. “Full food security” describes a household with very little trouble or anxiety regarding household members gaining access to food, while “marginal food security” refers to anxiety regarding household members gaining access to food, without a reduction in the quantity, quality, or the variety of foods consumed [7,8]. “Low food security” defines households that reduce the quality, variety, and desirability of foods, yet the quantity of foods consumed remains adequate [7,8]. “Very low food security” exists when the quantity of foods consumed is inadequate, and eating patterns of the household are subsequently disrupted [7,8]. Households that were considered to have full or marginal food security were classified as food secure (<3 affirmative responses); those with low or very low food security were classified as food insecure (≥3 affirmative responses) [9]. Household food security is reflective of conditions over the previous 12 months, that serve as the inherent reference period in the USDA’s Household Food Security Survey Module [10].

DS use in the previous 30 days was collected during the household interview via an in-home inventory and the dietary supplement questionnaire. Participants were asked to show interviewers the containers for all products taken in the past 30 days. For each DS reported, interviewers recorded the name, manufacturer, form of the products (e.g., tablet) and dose per serving for selected single nutrient products from the label. Detailed information on the consumption frequency, amount, and duration of DS use were also collected for each product reported. Mean daily nutrient intakes from supplemental sources for each individual were calculated using the total number of reported days, amount taken per day, and the dose per serving of each product from the label. More information on the NHANES DS component protocol can be found elsewhere [5,11,12,13]. All information from DS was obtained from the dietary supplement questionnaire in the in-home inventory.

Dietary intake was self-reported in the MEC using an in-person 24 h dietary recall. A second 24 h dietary recall was completed via telephone approximately 3–10 days after the MEC exam. Both 24 h recalls were collected by trained interviewers using the USDA’s automated multiple-pass method [14,15]. The USDA Food and Nutrient Database for Dietary Studies and the NHANES Dietary Supplement Database were used to convert foods, beverages, and DS as consumed to their respective nutrient values [16,17].

The micronutrients chosen for presentation in the main tables of this analysis were selected based on under-consumed micronutrients identified in the 2015–2020 Dietary Guidelines for Americans among some subgroups within the U.S. population: calcium, magnesium, iron, potassium, choline, folate and vitamins A, C, D, and E [1,2]. Micronutrients associated with lower intakes from diet alone among the food insecure (calcium, magnesium, zinc, and vitamins A and B6) in a systematic review were also included [4]. However, vitamins A and E were not available in the NHANES 2011–2014 DS data files; thus, total nutrient intakes could not be estimated, and intakes are reflective of food sources only for these vitamins in the supplementary material provided in Tables S1 and S2. Information on all of the additional micronutrients examined are provided in the supplementary material (Tables S1 and S2). It should be noted that the UL for folate only applies to the synthetic form, folic acid, obtained from DS and fortified foods. Thus, folic acid was the only form of folate used to estimate the proportion of the population exceeding the UL. Sodium was excluded since negligible amounts are found in DS [18].

An adaptation of the National Cancer Institute (NCI) method [19,20] was used to estimate (1) distributions of usual micronutrient intakes (from foods alone and total) by men and women and (2) the proportions of the subpopulations (i.e., sex, food security status) whose usual intakes were above or below age and sex-specific DRIs. The NCI method is used to estimate the distributions of “usual” or “long-term mean daily” intakes by accounting for random measurement error (i.e., within-person variation). It was adapted to estimate the contributions of DS to usual micronutrient intake estimates through the incorporation of reported DS intakes from the dietary supplement questionnaire, using the method described by Bailey et al. [18,21]. Covariates incorporated in the usual intake models included day of the week of the dietary recall (weekend/weekday), interview sequence (first or second dietary recall), and DS use overall. Categorical variables for sex and food security status were used for subgroup analyses. Mean daily nutrient intakes from DS and their relative contribution to total intakes were estimated by adding nutrients from supplemental sources to the adjusted distributions of usual intake from dietary sources to estimate the distributions of total usual micronutrient intake among the adult total population (DS users and nonusers combined) [18,22]. The relative contribution of DS to total micronutrient intakes was calculated by dividing the total usual micronutrient intake from DS by the total usual micronutrient intake from all sources (inclusive of foods and DS) at the population level (Table 1).

Total usual micronutrient intake distributions were compared to age and sex-specific DRIs established by the National Academies of Science, Engineering, and Medicine in order to compare total usual micronutrient intakes to the DRIs, including the %< Estimated Average Requirement (EAR), %> Adequate Intake (AI), and %> Tolerable Upper Intake Level (UL) using the cut-point method [23,24]. The EAR cut-point method assumes that the nutrient requirement distribution is symmetric; therefore, it cannot be applied to iron, since the requirement distribution for iron is skewed in reproductive-aged women [25]. Therefore, iron estimates are only presented relative to the EAR for men.

All statistical analyses were performed using SAS software (version 9.4; SAS Institute Inc., Cary, NC, USA) accounting for the NHANES complex survey design and sampling weights to adjust for differential non-response and non-coverage, and oversampling and post-stratification. Standard errors (SE) for all statistics of interest were approximated using Fay’s modified Balanced Repeated Replication technique [26,27]. Differences in the proportion of the population with total usual micronutrient intakes < EAR or > AI within sex groups by food security status were compared using pairwise t-tests; a Bonferroni- adjusted *p*-value of <0.005 was considered statistically significant (Table 2, Table S2). Multiple comparisons were conducted using a pairwise t statistic to assess differences in the proportion of U.S. adult supplement users with total usual micronutrient intakes > UL within sex groups by food security status; a Bonferroni-adjusted *p*-value of <0.0125 was considered statistically significant (Figure 2).

## 3. Results

In general, the proportion of nutrients from dietary sources was greater than the proportion from DS. However, the relative contributions of DS to total intake varied by nutrient, with the lowest contributions for choline, (0.5%), potassium (0.5%), phosphorus (0.5%), vitamin K (7.0%), and magnesium (8.2%) (Table 1, Table S1). DS contributed over half of total intake for vitamins B6 (61%), B12 (93%), C (52%), and D (71%). However, even with high intakes of vitamins C and D from DS, the proportion of adults at risk of inadequacy remains high (Table 1). Most notably, for vitamin D, 98% of women and 92% of men in the U.S. were at risk of inadequate intake from foods alone, yet, the prevalence of vitamin D inadequacy among adults ranged from 59% to 66%, depending on the sex, even when taking into account nutrient intakes from DS. Smaller differences were found for calcium, magnesium, and vitamin C in both men and women, and for zinc and vitamin B6 in women alone. Calcium DS reduced the prevalence of at-risk intakes from 26% (foods alone) to 21% (total) among men and from 58% (foods alone) to 41% (total) among women, although, unlike vitamin D, calcium from supplemental sources varied, accounting for only 4% to 21% of total intake depending on the sex/food security group considered.

**Figure 1:**
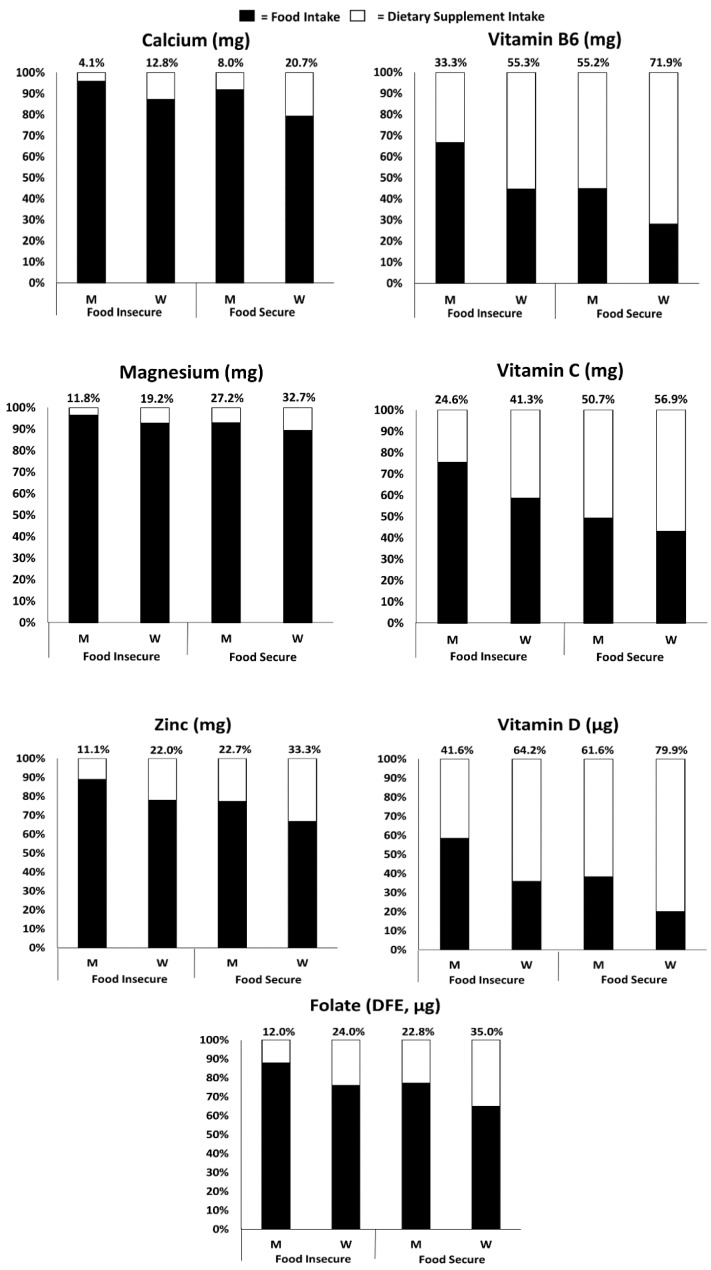
Relative contribution of foods/beverages and dietary supplements to total usual intakes for selected nutrients by age group among men and women by food security status (≥19 years) in the U.S., 2011–2014. ^1^ (^1^ The analytic sample includes individuals ≥19 years old that were not pregnant or lactating with complete information for food security and the day 1 and 2, 24 h dietary recalls. Percentages above each bar represent the relative contribution from dietary supplements). Abbreviations: M, Men; W, Women; DFE, Dietary Folate Equivalents.

DS contributed a larger proportion to total usual intakes among adults living in food secure households compared with those living in food insecure households for all nutrients examined (Figure 1). A higher prevalence of inadequate intakes was observed among adults living in food insecure than in food secure households, especially for magnesium, vitamin C, and vitamin D (Table 2). Adults living in food insecure households also had a lower prevalence of intakes exceeding the AI for potassium when compared with those in food secure households (Table 2). Similar patterns were observed for intakes of copper, niacin, riboflavin, vitamin B12, and vitamin K in men and women, and phosphorous, selenium, and zinc in men alone (Table S2).

**Figure 2:**
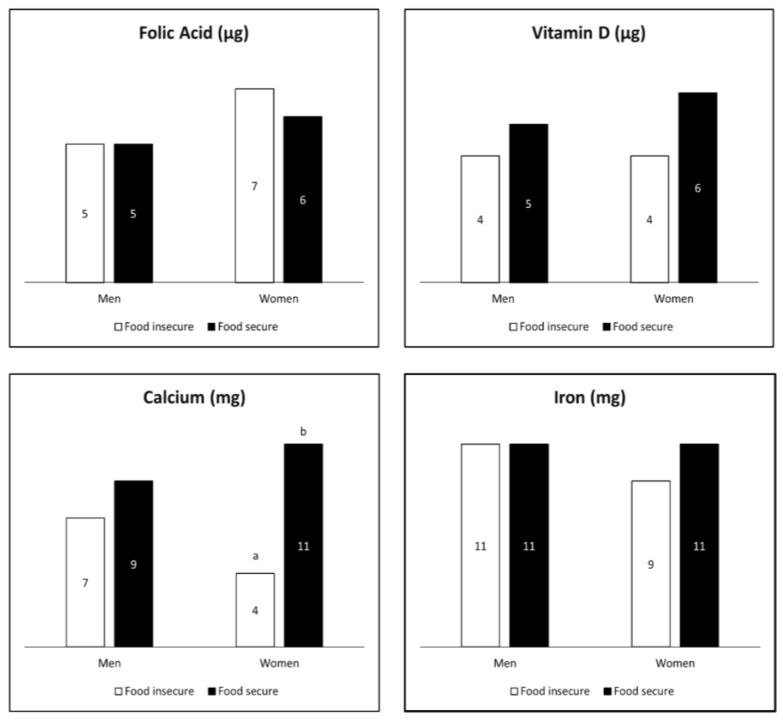
Estimated percent (%) of total micronutrient intakes above the Tolerable Upper Intake Level (UL) by food security status among adult (≥19 years) supplement users in the U.S., 2011–2014. ^1,2^ (^1^ The analytic sample includes individuals ≥19 years old that were not pregnant or lactating with complete information for food security and the day 1 and 2, 24 h dietary recalls. Numerical data labels within each bar represent the estimated proportion (%) of U.S. adult supplement users with intakes greater than the UL. ^2^ Different superscript letters denote a significant difference between food security categories within sex at a *p*-value < 0.0125.).

A small proportion of supplement users had total usual intakes that exceeded the UL for folic acid, vitamin D, calcium, or iron (Figure 2); but this was only significantly different for women by food security status with regard to calcium.

## 4. Discussion

Our findings suggest that DS aid in reducing the proportion of the population at risk for inadequate intakes, especially for vitamin D, calcium, and vitamin C. However, many Americans still have low intakes of some micronutrients, even with the use of DS, which is especially true among U.S. adults with food insecurity. While this study focuses on individual nutrients, the health effects of a diet, especially those related to chronic diseases, are determined by the sum and interaction of many food constituents in addition to nutrients. Many of these constituents are not yet fully understood and do not have DRIs. Plant products are particularly complex with many bioactive components (flavonoids, various types of fiber, etc.). The recognized health benefits of diets rich in fruits, vegetables, and whole grains, for example, are attributable to more than their nutrient content [1]. Consequently, the Dietary Guidelines for Americans focus on the quality of the overall dietary pattern. To address the problem of food insecurity, the 2015–2020 Dietary Guidelines for Americans call for supporting individuals in making healthy food choices by expanding nutrition-assistance programs and creating networks and partnerships to address the problem. One example is improving the offerings at food pantries [28]. Another is expanding programs, such as the Expanded Food and Nutrition Education Program and SNAP-Ed, which teach food resource management [29].

Two studies have confirmed that total usual nutrient intakes, inclusive of DS, vary by income alone using the family poverty-to-income ratio (PIR) [21,30]. However, as outlined by the Academy of Nutrition and Dietetics, food security results from a constellation of factors that may predispose individuals to nutrition risk beyond income alone, including environmental factors like transportation, food prices, housing costs, unemployment, and social capital, among others [31]. In a previous analysis in Canada, Kirkpatrick et al. revealed a higher prevalence of micronutrient inadequacy for several micronutrients among adolescents and adults living in food insecure as compared with food secure households, specifically for vitamin A, thiamin, riboflavin, vitamin B6, folate, vitamin B12, magnesium, phosphorus, and zinc [32]. The present analysis extends prior foods-based work by examining total micronutrient intakes, inclusive of DS, by food security status. Adults living with food insecurity had a higher risk of micronutrient inadequacy for most micronutrients examined, except for calcium, iron (examined only in men), folate, or choline, when compared with food secure adults. Most notably, in addition to the micronutrients identified by Kirkpatrick et al. [32], the present analysis observed a higher prevalence of micronutrient inadequacy among adults living with food insecurity for copper, potassium, niacin, and vitamins C, D, E, and K in both men and women, and selenium in men alone. While nutrient inadequacy was more of a concern among adults living in food insecure households, all DS users, regardless of food security status, had an increased likelihood of usual intakes above the UL for iron, calcium, vitamin D, and folic acid, increasing risk of adverse health effects [25,33,34].

A strength of this analysis is that the models applied to examine total usual intakes adjust for the effects of random measurement error to the extent possible, in addition to using the recommended method of adding mean daily nutrient intakes from supplemental sources to the adjusted usual nutrient intakes from dietary sources [18,22]. USDA’s automated multiple-pass method is a state-of-the-art method for capturing dietary data, as is the Food and Nutrient Database for Dietary Studies that supports it. However, self-reported dietary data are prone to systematic measurement error, like energy underreporting, that may result in an underestimation of micronutrient intakes from the diet. Furthermore, the analysis of nutrients in DS relies on label declarations on products, rather than analytically derived values, that are likely to result in an underestimation of micronutrients from DS [35]. Furthermore, we assume that the DS intake reported for the past 30 days during the in-home interview reflects long-term, habitual DS intake, but little is known about the measurement error structure of DS reporting [18]. NHANES is a nationally representative survey of the U.S. noninstitutionalized population. However, the response rates for the years 2011–2012 and 2013–2014 for adults were 66% and 65%, respectively [36,37], and total usual nutrient intakes could not be estimated for vitamins A and E, as these nutrients are not included in the current NHANES DS data files. An additional limitation is that the bioavailability of nutrients from DS compared to the bioavailability of nutrients from foods remains largely unknown.

## 5. Conclusions

In summary, our findings are consistent with previous reports that demonstrate that many U.S. adults have inadequate intakes of potassium, magnesium, calcium, vitamin D, and/or vitamin C, even with the use of DS, and that those living with food insecurity have a higher prevalence of micronutrient risk than those not living with food insecurity. These findings suggest that, while DS can be helpful in meeting nutrient requirements for adults for some nutrients, potential excess may also be of concern for certain nutrients among supplement users.

## Figures and Tables

**Table 1:** Relative contribution of dietary supplements to total usual nutrient intakes and the estimated percent (%) of usual intakes (foods alone and total) below the Estimated Average Requirement or above the Adequate Intake for select nutrients among adults (≥19 years) in the U.S., 2011–2014. ^1^

	All Adults		Men			Women		
	Total Usual Nutrient Intakes		Total Usual Nutrient Intakes		Usual Intake from Foods	Total Usual Nutrient Intakes		Usual Intake from Foods
	% Contribution from DS	%<EAR/>AI (SE)	% Contribution from DS	%<EAR/>AI (SE)	%<EAR/>AI (SE)	% Contribution from DS	%<EAR/>AI (SE)	%<EAR/>AI (SE)
Calcium (mg)	13.2%	31.0 (1.0)	7.5%	21.0 (1.0)	26.0 (1.2)	19.5%	41.0 (1.3)	58.0 (1.6)
Iron (mg) ^2^	16.9%	--	8.4%	0.1 (0.1)	0.2 (0.1)	25.3%	--	--
Magnesium (mg)	8.2%	45.2 (1.0)	6.6%	46.0 (1.2)	52.0 (1.3)	10.1%	43.6 (1.2)	50.7 (1.3)
Potassium (mg) ^3^	0.5%	37.0 (1.0)	0.5%	36.0 (1.3)	35.0 (1.3)	0.5%	33.0 (1.5)	33.0 (1.6)
Zinc (mg)	26.0%	16.8 (0.7)	21.0%	12.7 (1.1)	16.3 (1.4)	32.1%	13.2 (1.1)	17.3 (1.2)
Choline (mg) ^3^	0.5%	12.3 (0.6)	0.3%	12.0 (1.1)	11.7 (1.1)	0.5%	3.6 (0.7)	3.4 (0.7)
Folate (DFE, µg) ^4^	27.2%	9.0 (0.8)	21.4%	5.0 (0.6)	6.0 (0.8)	33.4%	12.0 (1.2)	15.9 (1.6)
Vitamin B6 (mg)	61.4%	6.2 (0.6)	52.7%	1.9 (0.5)	2.6 (0.6)	71.2%	10.6 (0.8)	14.4 (1.0)
Vitamin C (mg)	51.7%	35.0 (1.1)	48.1%	39.0 (1.7)	50.8 (1.7)	55.0%	32.0 (1.2)	44.0 (1.4)
Vitamin D (µg)	70.6%	63.1 (0.7)	59.8%	66.4 (1.0)	91.5 (0.9)	78.2%	59.1 (1.2)	98.4 (0.3)

Abbreviations: DS, dietary supplement; EAR, Estimated Average Requirement; AI, Adequate Intake; SE, standard error. ^1^ The analytic sample includes individuals ≥19 years old that were not pregnant or lactating with complete information for the day 1 and 2, 24 h dietary recalls. ^2^ Proportion of the population below the EAR for iron was unable to be assessed using the cut-point method in women due to a skewed distribution of nutrient requirements. ^3^ Indicates % > AI rather than % < EAR. This occurs when sufficient scientific evidence is not available to establish an EAR. ^4^ As dietary folate equivalents (DFEs). 1 DFE = 1 µg food folate = 0.6 µg of folic acid from fortified food or as a supplement consumed with food = 0.5 µg of a supplement taken on an empty stomach.

**Table 2:** Proportion of the population falling below the Estimated Average Requirement or above the Adequate Intake from total usual nutrient intakes of select nutrients, by food security status, among adults (≥19 years) in the U.S., 2011–2014. ^1,2^

	Food Security Status, % < EAR/> AI (SE)	
	Food Insecure	Food Secure
Men (n, %)	(915, 14.9%)	(3993, 85.1%)
Calcium (mg)	25.0 (2.2)	20.0 (1.3)
Iron (mg) ^3^	0.3 (0.2)	0.1 (0.1)
Magnesium (mg)	57.2 (1.7) ^a^	43.9 (1.5) ^b^
Potassium (mg) ^4^	25.0 (2.5) ^a^	37.0 (1.6) ^b^
Zinc (mg)	20.1 (2.4) ^a^	11.3 (1.1) ^b^
Choline (mg) ^4^	12.6 (2.3)	11.8 (1.1)
Folate (DFE, µg) ^5^	7.0 (1.8)	4.0 (0.7)
Vitamin B6 (mg)	6.1 (1.3) ^a^	1.4 (0.4) ^b^
Vitamin C (mg)	49.0 (3.8) ^a^	37.0 (1.7) ^b^
Vitamin D (µg)	79.2 (1.5) ^a^	64.1 (1.2) ^b^
Women (n, %)	(1010, 15.6%)	(4034, 84.4%)
Calcium (mg)	46.0 (3.0)	40.0 (1.3)
Iron (mg) ^3^	--	--
Magnesium (mg)	56.9 (2.6) ^a^	40.9 (1.4) ^b^
Potassium (mg) ^4^	24.0 (3.1) ^a^	35.0 (1.7) ^b^
Zinc (mg)	17.1 (2.8)	12.2 (1.3)
Choline (mg) ^4^	4.4 (1.5)	3.5 (0.6)
Folate (DFE, µg) ^5^	15.0 (3.5)	11.0 (1.3)
Vitamin B6 (mg)	19.0 (1.6) ^a^	8.8 (0.9) ^b^
Vitamin C (mg)	42.0 (2.4) ^a^	29.0 (1.3) ^b^
Vitamin D (µg)	74.7 (1.7) ^a^	56.2 (1.2) ^b^

Abbreviations: EAR, Estimated Average Requirement; AI, Adequate Intake; SE, standard error. ^1^ The analytic sample includes individuals ≥19 years old that were not pregnant/lactating with complete information for the day 1 and 2, 24 h dietary recalls. ^2^ Different superscript letters denote a significant difference between food security categories at a *p*-value < 0.005. ^3^ Proportion of the population below the EAR for iron was unable to be assessed using the cut-point method in women due to a skewed distribution of nutrient requirements. ^4^ Indicates %> AI rather than %< EAR. This occurs when sufficient scientific evidence is not available to establish an EAR. ^5^ As dietary folate equivalents (DFEs). 1 DFE = 1 µg food folate = 0.6 µg of folic acid from fortified food or as a consumed with food = 0.5 µg of a supplement taken on an empty stomach.

## Supplementary Materials

The following are available online at https://www.mdpi.com/2072-6643/12/1/38/s1, Table S1: Relative contribution of dietary supplements to total usual nutrient intakes and the estimated percent (%) of usual intakes (foods alone and total) below the Estimated Average Requirement or above the Adequate Intake for other nutrients among adults (≥19 years) in the U.S., 2011–2014, Table S2: Proportion of the population falling below the Estimated Average Requirement or above the Adequate Intake from total usual nutrient intakes of other nutrients, by food security status among adults (≥ 19 years) in the U.S., 2011–2014.
