# Supplementary material for: Total Usual Micronutrient Intakes Compared to the Dietary Reference Intakes among U.S. Adults by Food Security Status
Source: Nutrients. 2019 Dec 22;12(1):38. doi: 10.3390/nu12010038 (PMC7019721; doi:10.3390/nu12010038)
Supplement: Supplementary file 1 [file nutrients-12-00038-s001.pdf]

Table S1. Relative contribution of dietary supplements to total usual nutrient intakes and the estimated percent (%) of usual intakes (foods alone and total) below the Estimated Average Requirement or above the Adequate Intake for other nutrients among adults (≥19y) in the U.S., 2011-2014[1].

	All Adults		Men			Women		
	Total Usual Intakes		Total Usual Intakes		Usual Intake from Foods	Total Usual Intakes		Usual Intake from Foods
	**% Contribution from DS**	**%<EAR/>AI (SE)**	**% Contribution from DS**	**%<EAR/>AI (SE)**	**%<EAR/>AI (SE)**	**% Contribution from DS**	**%<EAR/>AI (SE)**	**%<EAR/>AI (SE)**
Copper (mg)	18.4%	5.3 (0.4)	11.8%	2.2 (0.4)	2.5 (0.4)	21.4%	8.5 (0.7)	10.3 (0.7)
Phosphorous (mg)	0.5%	1.0 (0.1)	0.4%	1.0 (0.2)	1.0 (0.2)	0.5%	0.0 (0.1)	0.0 (0.0)
Selenium(μg)	13.5%	0.1 (0.0)	11.8%	0.0 (0.0)	0.0 (0.0)	15.7%	0.8 (0.2)	1.0 (0.2)
Niacin (mg)	26.7%	1.3 (0.2)	22.2%	0.2 (0.1)	0.2 (0.1)	32.2%	1.5 (0.2)	1.9 (0.3)
Riboflavin (mg)	51.1%	3.1 (0.3)	46.8%	1.9 (0.3)	2.2 (0.3)	57.1%	2.3 (0.3)	2.8 (0.4)
Thiamin (mg)	75.4%	5.7 (0.5)	71.6%	2.1 (0.4)	2.6 (0.5)	77.8%	7.0 (0.9)	9.3 (1.1)
Vitamin A (μg RAE)[2]	--	45.0 (1.7)	--	--	48.0 (2.3)	--	--	41.0 (1.3)
Vitamin B12 (μg)	92.8%	3.0 (0.4)	88.7%	1.0 (0.3)	1.1 (0.4)	95.4%	5.0 (0.6)	6.7 (0.8)
Vitamin E (mg ATE)[3]	--	79.6 (1.0)	--	--	70.5 (1.4)	--	--	88.2 (1.1)
Vitamin K (μg)[4]	7.0%	55.1 (1.3)	5.2%	45.9 (1.7)	41.6 (1.7)	8.5%	63.0 (1.8)	59.0 (2.0)

[1] The analytic sample includes individuals ≥19 years old that were not pregnant or lactating with complete information for the day 1 and 2, 24-hour dietary recalls.
[2] As retinol activity equivalents (RAEs). 1 RAE = 1 mg retinol, 12 mg b-carotene, 24 mg a-carotene, or 24 mg b-cryptoxanthin. Usual intakes < EAR are from food sources only.
[3] As a-tocopherol equivalents (ATEs). a-Tocopherol includes RRR-a-tocopherol, the only form of a-tocopherol that occurs naturally in foods, and the 2R-stereoisomeric forms of a-tocopherol (RRR-, RSR-, RRS-, and RSS-a-tocopherol) that occur in fortified foods and supplements. Usual intakes < EAR are from food sources only.
[4] Indicates % > AI rather than % < EAR. This occurs when sufficient scientific evidence is not available to establish an EAR.

Table S2. Proportion of the population falling below the Estimated Average Requirement or above the Adequate Intake from total usual nutrient intakes of other nutrients, by food security status among adults (≥ 19 y) in the U.S., 2011-2014[1,2]

	**Food Security Status, % < EAR / > AI (SE)**	
	**Food Insecure**	**Food Secure**
Men		
Copper (mg)	5.3 (1.2)[a]	1.6 (0.4)[b]
Phosphorous (mg)	1.0 (0.3)[a]	0.0 (0.0)[b]
Selenium (µg)	0.3 (0.2)[a]	0.0 (0.0)[b]
Niacin (mg)	0.8 (0.4)[a]	0.1 (0.0)[b]
Riboflavin (mg)	4.7 (1.2)[a]	1.4 (0.3)[b]
Thiamin (mg)	4.6 (1.4)	1.7 (0.4)
Vitamin A (µg RAE)[3]	60.0 (2.5)[a]	35.0 (2.0)[b]
Vitamin B12 (µg)	3.5 (1.3)[a]	1.0 (0.2)[b]
Vitamin E (mg ATE)[4]	78.1 (2.9)[a]	58.6 (1.5)[b]
Vitamin K (µg)[5]	18.5 (3.5)[a]	49.6 (1.9)[b]
Women		
Copper (mg)	14.9 (1.9)[a]	7.1 (0.7)[b]
Phosphorous (mg)	2.0 (0.8)	1.0 (0.2)
Selenium (µg)	1.5 (0.6)	0.7 (0.2)
Niacin (mg)	3.7 (1.0)[a]	1.2 (0.2)[b]
Riboflavin (mg)	4.2 (1.1)[a]	1.9 (0.3)[b]
Thiamin (mg)	9.4 (2.8)	6.4 (0.9)
Vitamin A (µg RAE)[3]	43.0 (3.2)[a]	28.0 (1.4)[b]
Vitamin B12 (µg)	9.0 (1.5)[a]	4.0 (0.6)[b]
Vitamin E (mg ATE)[4]	88.8 (1.8)[a]	74.6 (1.3)[b]
Vitamin K (µg)[5]	44.7 (2.7)[a]	66.3 (1.9)[b]

Abbreviations: Estimated Average Requirement; AI, Adequate Intake; SE, standard error.

[1] The analytic sample includes individuals ≥19 years old that were not pregnant/lactating with complete information for the day 1 and 2, 24-hour dietary recalls.

[2] Different superscript letters denote a significant difference between food security categories at a *p*-value < 0.005.

[3] As retinol activity equivalents (RAEs). 1 RAE = 1 mg retinol, 12 mg b-carotene, 24 mg a-carotene, or 24 mg b-cryptoxanthin. Usual intakes < EAR are from food sources only.

[4] As a-tocopherol equivalents (ATEs). a-Tocopherol includes RRR-a-tocopherol, the only form of a-tocopherol that occurs naturally in foods, and the 2R-stereoisomeric forms of a-tocopherol (RRR-, RSR-, RRS-, and RSS-a-tocopherol) that occur in fortified foods and supplements. Usual intakes < EAR are from food sources only.

[5] Indicates %> AI rather than %< EAR. This occurs when sufficient scientific evidence is not available to establish an EAR
